# The American Institute of Homœopathy Goes to Paris in 1900

**Published:** 1899-11

**Authors:** John B. Garrison

**Affiliations:** No. 111 East 70th Street, New York, N. Y.


### THE AMERICAN INSTITUTE OF HOMŒOPATHY GOES TO PARIS IN 1900.

### A TOUR THROUGH EUROPE IN COMPANY WITH FRIENDS.

A trip abroad is always interesting, but when arranged so as to avoid the worry incident to the care of baggage, seeing to tickets, securing lodging and that greatest of all nuisances—the giving of tips—the whole time from home may be one round of pleasure. This has been so arranged by the committee of arrangements appointed by the Institute, and the party, limited to homœopathic physicians, their families, and friends, will have the rare fortune of being in the care of Dr. Howard S. Paine, who, for fourteen years, has personally conducted parties of tourists through Europe, and he will see that travel is first-class in every respect—Steamships, Railways, and Hotels. ALL NECESSARY EXPENSES, including carriage drives, fees on land and admission to all places of interest, are included in the rates quoted, and are from New York to New York. Every thing of interest along the various lines will be visited. London, Paris, and all the principal cities will be intelligently explored, giving most time to the things of greatest interest. All night travel will be avoided and the Sabbath will be a day of rest. THOSE INTENDING TO MAKE THE TRIP MUST APPLY BY DECEMBER 15th, or at the very latest, by Jan. 1st, 1900, as the steamship companies refuse the ordinary favors of holding berths to near sailing time this season, on account of the heavy travel anticipated. *Commence talking about going right away!*

Six tours, designated "A," "B," "C," "D," "E," "F," all arranged so that they will be in Paris for the meeting of the INTERNATIONAL HOMŒOPATHIC CONGRESS,

July 18th, 19th, 20th, 21st, 1900, are graded in prices to suit the tastes and means of every one.

Tour "A."—Eighty days, costing $635, leaving New York June 27th, and arriving home Sept. 15th; visiting: Antwerp, Brussels, Paris, Versailles, Geneva, Lake Geneva, Castle of Chillon, Berne, Lake Thun, Interlaken, Lauterbrunnen, Staubbach Falls, Lake Brienz, Brunig Pass, Lake of the Four Cantons, Lucerne, Menaggio, Lake Como, Milan, Pisa, Rome, Florence, Venice, Brenner Pass, Bozen, Munich (trip to Ober-Ammergau optional), Dresden, Berlin, Potsdam, Frankfort, Wiesbaden, Biebrich, The Rhine, Cologne, Antwerp, Harwich, London, Stratford-on-Avon, Warwick, Kenilworth, Leamington, Southampton.

Tour "B."—Sixty-six days, costing $565, sailing same date as tour "A," and arriving home Sept. 1st. Visiting: The same as tour "A," as far as Bozen, then Innsbruck, Constance, The Black Forest, Heidelberg, Frankfort, Wiesbaden, Biebrich, The Rhine, Cologne, Antwerp, Harwich, London, Stratford-on-Avon, Warwick, Kenilworth, Leamington, Southampton.

Tour "C."—Fifty-three days, costing $435, sailing from New York same as "A" and "B," arriving home Aug. 18th. Visiting same as the two previous tours as far as the Rigi, then Neuhausen, The Falls of the Rhine, Heidelberg, Wiesbaden, Biebrich, The Rhine, Cologne, Brussels, Ostend, London, Warwick, Stratford-on-Avon, Kenilworth, Leamington, Southampton.

Tour "D."—Forty-nine days, costing $405. Sailing from New York June 20th, arriving in New York Aug. 7th. Visiting: Southampton, Salisbury, Stonehenge, Winchester, Cambridge, Peterborough, Ely, Lincoln, Doncaster, Ripon, Fountains Abbey, Durham, Melrose, Abbottsford, Edinburg, Sterling, Trossachs, Loch Lomond, Loch Katrine, Glasgow,

Keswick, Ambleside, Windemere, Chester, Stratford-on-Avon, Leamington, Kenilworth, Warwick, London, New Haven, Dieppe, Paris, Brussels, Antwerp.

Tour "E."—Thirty-seven days, costing $270. Sailing from New York July 7th, arriving in New York Aug. 12th. Visiting: Boulogne-sur-Mere, Paris, Versailles, Dieppe, New Haven, London, Warwick, Kenilworth, Stratford-on-Avon, Leamington, Chester, Glasgow.

Tour "F."—This tour of Denmark, Norway (North Cape), Sweden, and Russia is not, at present writing, entirely completed as to details, but will offer a most delightful trip for those who have already visited England and the Continent, and particulars will be given in a short time.

In securing Dr. Paine to assume the management of these tours we have a guarantee that members will be taken care of in a thoroughly first-class manner, even in Paris, where those having less experience would have difficulty in securing good accommodations during the Exposition. Do not delay in making your arrangements to go, for already some steamship lines are making many bookings. Ten have already signified their intentions of going. A circular, giving a complete itinerary, with dates of visits and length of time spent at each place, will soon be ready for distribution and will be sent to all who desire to look the matter up.

*Note.*—The rates quoted are subject to modification according to location of the room and number of persons occupying the room.

Address all inquiries to the chairman,

Dr. John B. Garrison,

No. 111 East 70th Street,

New York, N. Y.

